# Application of Artificial Intelligence in Orthognathic Surgery: A Scoping Review

**DOI:** 10.1155/bmri/8284581

**Published:** 2025-06-12

**Authors:** Saeed Reza Motamedian, Sadra Mohaghegh, Maral Niazmand, Hossein Mohammad-Rahimi, Nima Ahmadi, Mina Yaseri, Helia Sadat Haeri Boroojeni

**Affiliations:** ^1^Department of Orthodontics, School of Dentistry, Shahid Beheshti University of Medical Sciences, Tehran, Iran; ^2^Department of Oral and Maxillofacial Surgery, School of Dentistry, University of Nevada, Las Vegas, Nevada, USA; ^3^Department of Oral and Maxillofacial Surgery, School of Dentistry, Shahid Beheshti University of Medical Sciences, Tehran, Iran; ^4^Department of Dentistry and Oral Health, Aarhus University, Aarhus, Denmark; ^5^Department of Oral and Maxillofacial Surgery, Shahid Beheshti University of Medical Sciences, Tehran, Iran

**Keywords:** artificial intelligence, machine learning, neural networks, orthognathic surgery, review

## Abstract

**Objective:** This study was aimed at reviewing the application of different artificial intelligent algorithms used in different phases of orthognathic surgeries, which include diagnosis, treatment planning, soft tissue prediction, outcome evaluation, and complication assessment. This aimed to update clinicians on this technology to integrate it into their decision-making, in addition to being aware of its challenges and potential areas for further assessment.

**Materials and Methods:** Electronic search was done in PubMed, Scopus, Embase, and Cochrane databases. Studies that reported the application of artificial intelligence (AI) in different aspects of orthognathic surgery were included.

**Results:** From 656 studies, a total of 29 articles met the inclusion criteria and were used to categorize the application of AI as follows: (1) Diagnosis in which studies showed the sensitivity of 75%–95.5% for specifying the need for orthognathic surgery; (2) treatment planning in which AI was used for osteotomy design and bony reference point determination with 3.99–4.73 mm of error; (3) soft tissue prediction in which AI models showed a success rate of 64.3%–100%; (4) outcome evaluation in which AI was used to assess the impact of surgery on asymmetry, facial attractiveness, and esthetic improvements quantitatively; and (5) complication assessment with an accuracy of 98.7% for predicting postsurgical systemic infection and 7.4 mL of error for blood loss.

**Conclusion:** AI can be potentially considered as a proper alternative to conventional approaches to fasten the procedure related to orthognathic surgery and with comparable accuracy to conventional methods.

## 1. Introduction

**Figure 1:**
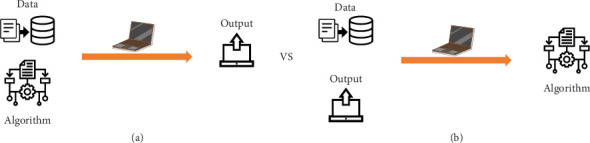
Comparison of (a) conventional data processing and (b) using machine for data processing. In conventional programming, an algorithm is given to the computer, and output is being produced by applying the algorithm to input (dataset). But in machine learning, input and output are given to the computer as “ground truth” to teach the computer, and further unspecified datasets are being predicted based on it.

John McCarthy first described artificial intelligence (AI) in 1958 as the science and engineering of making intelligent machines [1]. Since then, AI has evolved significantly, making profound impacts across various fields. Today, AI aims to implement machines and algorithms to imitate humanoid intelligence to do tasks that humans routinely do [2, 3] (Figure 1). It has spread worldwide, finding applications in diverse fields such as engineering, information technologies, language translations, economics, and governing analysis [4–6].

In medicine, AI has shown great potential, with studies highlighting its applications in diagnosis, treatment planning, follow-ups, outcome assessments, and prognosis predictions [6–8]. Notably, AI research has made significant strides in areas such as cancer, neurology, and cardiovascular diseases [7]. In dentistry, AI has proven feasible for diagnosing tumors, caries detection, diagnostic imaging, periapical disease classifications, and cephalometric spotting [6].

**Figure 2:**
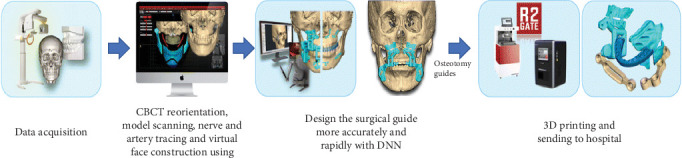
Schematic workflow of using AI in orthognathic surgery. This figure only shows AI application in treatment planning. Meanwhile, this technology can be used in other fields of surgery such as outcome evaluation and soft tissue prediction.

Orthognathic surgery is a complicated and multidisciplinary surgery of jaws that enrolls orthodontists and oral and maxillofacial surgeons, which also benefits from AI [3]. AI helps minimize personal bias and technique sensitivity in diagnosing and treatment planning by interpreting clinical and paraclinical data accurately and quickly (Figure 2) [6, 9]. Research has shown that AI models can significantly aid in diagnosing, decision-making, and treatment planning in orthognathic surgeries.

Machine learning (ML), a key subdivision of AI, plays a pivotal role in medical sciences. ML involves data-driven AI [10], where a computer learns inputs and outputs rapidly to develop predictive rules [11]. Datasets are being included in the ML algorithm in which ground truths of the task have been previously specified [10, 12]. To input datasets, they are split into training, validation, and test sets. The training set is given to the machine, and an algorithm is developed based on it to foresee future test datasets [13–15]. Meanwhile, the validation set evaluates the algorithm's generalization by imitating the test dataset and tuning the model [14].

**Figure 3:**
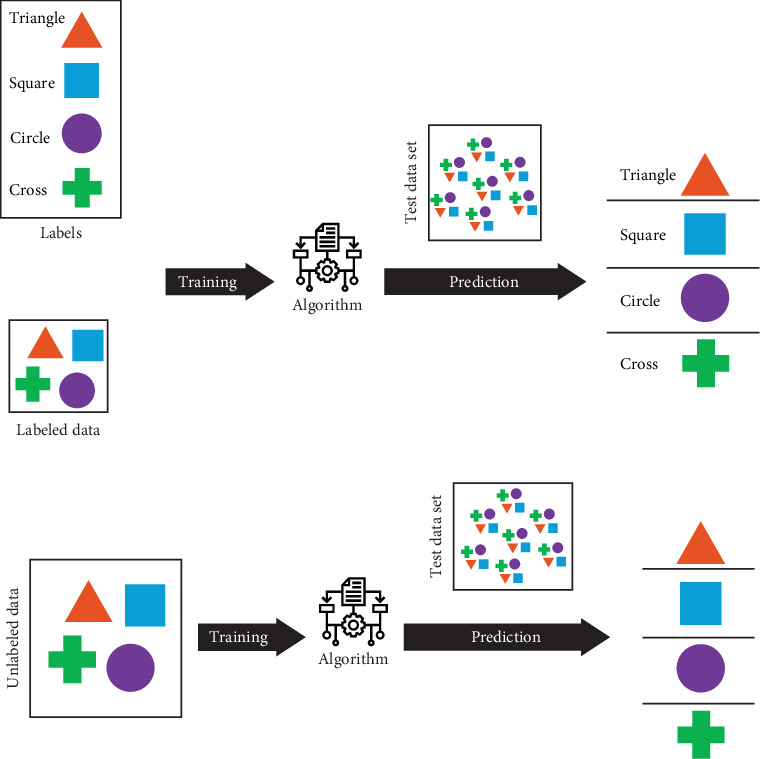
Comparison of (a) supervised and (b) unsupervised methods. In supervised learning, training datasets are given to the computer with a label and specified classification, and the computer distinguishes test datasets and categorizes them based on this specification. In contrast, training datasets are given to an unsupervised learning machine, and it tries to discover the pattern that lies in the datasets by itself and categorize test datasets based on them.

**Figure 4:**
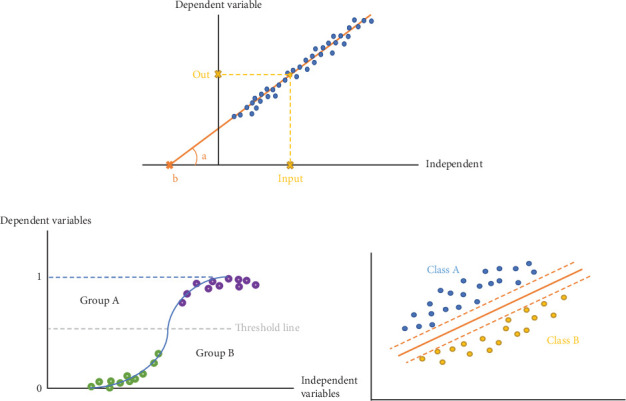
Different types of regression analyses. Regression is the act of predicting dependent variable by developing an equation. (a) Linear regression aims to define a relationship between independent and dependent variables by determining *a* and *b* in *y* = *ax* + *b*. In this diagram, red line is produced by machine using training datasets (blue points) and test dataset (yellow point) is placed in the diagram based on its independent variable (input). Dependent variable (output) is being predicted based on the line. *a* and *b* are mentioned in the diagram. (b) For classifying datasets into two groups, groups are considered 0 and 1 in diagram, and a threshold is being defined to put datasets in a distinct class. (c) Support vector machine: to classify datasets by defining an optimal borderline between them.

ML encompasses supervised and unsupervised learning [14] (Figure 3). In supervised learning, training datasets are given to the computer with a label and specified classification, and the computer distinguishes test datasets and categorizes them based on this specification. In contrast, training datasets are given to unsupervised learning machines, and they try to discover the pattern that lies in the datasets by themselves and categorize test datasets based on them. Supervised learning includes tasks like regression and classification. Regression is the task of predicting a continuous dependent quantity by developing an equation, such as using linear regression to predict blood loss during surgery [16], whereas classification refers to anticipating the labels of the imported data and categorizing datasets (Figure 4). For example, classifying datasets of patients with Class III malocclusion into three leading subtypes to determine their treatment consequences [17]. Unsupervised learning includes clustering, grouping datasets based on similarities without predefined labels [14].

As mentioned before, the commonest regression algorithm is linear regression. Linear regression is the most straightforward algorithm, showing the linear relationship between dependent and independent variables using the *y* = *ax* + *b* equation by considering dependent variables such as blood loss prediction during surgery as *y* and independent variables like actual blood loss as *x*. The machine's job is to recognize *a* and *b* and foresee actual blood loss in other cases based on them [14] (Figure 4).

Logistic regression and support vector machine (SVM) are algorithms that implement (purpose) classification to solve problems. Logistic regression is used for one binary dependent and several independent variables like the need for surgery in cleft palate in mixed dentition and prognostic factors such as age and type of cleft. Logistic regression applies *y* = 1/1 + *e*^−(*ax* + *b*)^ to create a sigmoidal curve where *y* values are placed between 0 and 1 [16, 18, 19] (Figure 4). SVM can also be used for classification by defining a specific border between variables. For instance, one of the possible procedures is that the machine receives the patients' radiographic data, defines the threshold of decision-making, and categorizes them into those who require surgery and those who do not. SVM is practical in limited sample sizes [20, 21].

**Figure 5:**
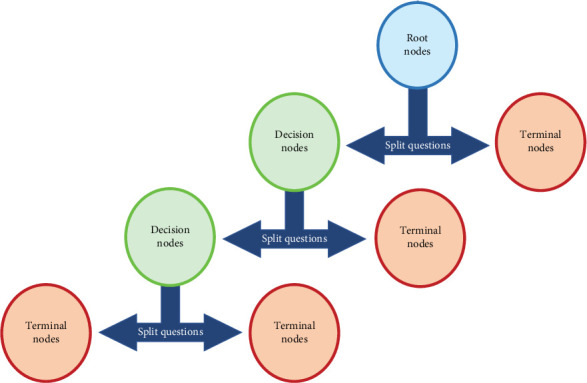
Decision tree schematic diagram.

Decision trees, which can be used for both regression and classification, are another subset of the supervised method. They consist of a combination of initial datasets (root nodes) and questions to help categorize imported data (decision nodes) to define the final subgroups (terminal nodes) (Figure 5). Dataset splitting is initiated at the root node and attaches to either a decision node for further splitting or a terminal node to predict the data category, which will continue to reach a plausible terminal node for datasets [14]. Different trees could be considered altogether since a large number of inaccurate decision trees lead to a more accurate answer. Indeed, decision trees with different guiding factors can be created, and the final output is the most selected answer of all trees [22, 23]. Random forest is one of the extensions of decision trees and has been used to predict the need for future orthognathic surgery in cleft patients. This procedure receives the influencing factors based on the previous researches and uses them as splitting questions until reaching the next decision node or the terminal node.

**Figure 6:**
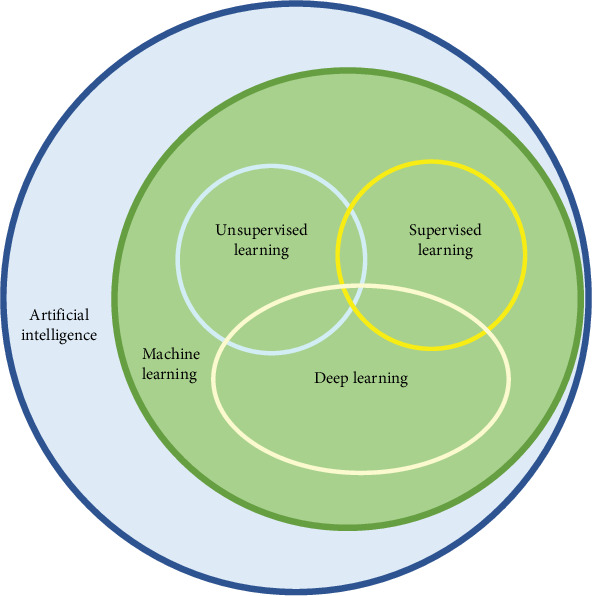
Subsets of AI models.

**Figure 7:**
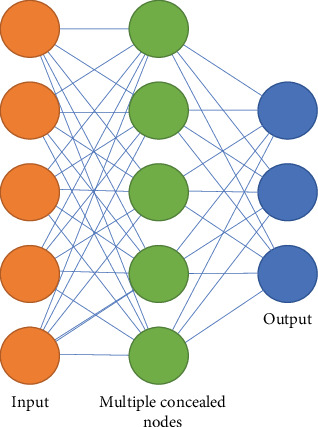
Artificial neural network schematic diagram. Deep learning is a subset of machine learning algorithms that aims to process input datasets, using artificial neural network architectures, in several hidden layers of the machine. Datasets are eventually given as output.

Deep learning is a vast ML subcategory with different applications in the medical field. It can hold a big deal of data and learn features for future predictions without requiring manual feature extraction [14, 24] (Figure 6). There are three typical structures of deep learning in the medical realm. First, artificial neural networks (ANNs) are based on biological neural networks and consist of artificial nodes, standing in different layers such as input, output, and some hidden nodes between them, which are connected by artificial axons and dendrites (Figure 7) [14, 24]. For example, to define the best place for an osteotomy cut, cone beam computed tomography (CBCT) and cast models are fabricated and imported to a network (nonequivalent point network), and nerves and arteries are traced, and the best osteotomy cut is defined to design a surgery guide template based on it [25, 26]. ANN analyzes the imported images pixel by pixel independently. Therefore, the relation between the pixels is not considered. CNN has emerged as a modification of ANN to not only analyze each pixel but also consider the relation between them. Nowadays, this is one of the commonest methods widely used as a substitution for conventional ANN for image analysis [24].

The following study is aimed at reviewing the application of different AI algorithms used in different phases of orthognathic surgeries, covering diagnosis, treatment planning, soft tissue prediction, outcome evaluation, and complication assessment. By doing so, it seeks to update clinicians on this evolving technology and let them integrate it into their daily decision-making. Also, technological challenges and requirements, in addition to research gaps for further assessments, are being specified.

## 2. Methods and Materials

### 2.1. Protocol and Registration

This systematic review was performed according to the Joanna Briggs methodology for scoping reviews (JBI) [27, 28]. The initial research question based on the PCC format was:

Population (P): patients undergoing orthognathic surgery.

Concept (C): AI algorithms (both ML and deep learning).

Context (C): different phases of orthognathic surgeries such as diagnosis, treatment planning, soft tissue prediction, outcome evaluation, and complication assessment.

### 2.2. Eligibility Criteria

Inclusion criterion was as follows:

1. Studies that assessed the function of the AI models used in diagnosis, treatment planning, outcome evaluation, and other aspects of orthognathic surgery

Exclusion criteria were as follows:

1. Studies that used AI for problems not related to orthognathic surgery

This exclusion criterion ensures that the review focuses solely on the application of AI within the specific domain of orthognathic surgery. Including studies addressing unrelated problems would dilute the review's focus and potentially introduce irrelevant findings, making it difficult to draw meaningful conclusions about the specific impact of AI in this surgical field.

2. Studies that used technologies other than AI

This criterion emphasizes the review's specific interest in AI technologies. Including studies using other technologies (e.g., traditional image analysis and non-AI–based prediction models) would broaden the scope beyond the targeted area of AI application.

3. Review articles

Review articles offer a broad overview of a topic but often lack the primary data and specific methodological details needed for a systematic review. Including review articles would introduce a level of indirectness in the analysis, making it difficult to assess the direct impact of AI in orthognathic surgery based on secondary sources.

4. Book sections and thesis studies

Book sections and theses, while potentially valuable, often lack the rigorous methodology and reporting standards typically found in peer-reviewed journal articles. Their inclusion could compromise the quality and reliability of the systematic review.

### 2.3. Information Sources and Search Strategies

An electronic search was performed in PubMed, Scopus, Embase, Cochrane, IEEE Xplore, and ACM digital library databases to find pertinent articles. Search queries mentioned in Table 1 were used. English articles published before June 2022 were considered. The reference list of the included studies and related reviews was also screened for possible related articles.

### 2.4. Study Selection and Data Collection Process

Five investigators (S.M., M.N., N.A., H.S.H.B., and M.Y.) screened the articles. Duplicate studies were removed by importing the references into EndNote (Thomson Reuters, Toronto, Canada). Study selection and data extraction were performed independently, and independent experts (S.R.M. and H.M.-R.) resolved any conflicts. Each article was screened by two of the investigators. After choosing the articles based on their titles and abstracts, full-text analyses were performed. Four investigators (S.M., N.A., M.N., and M.Y.) performed data extraction, and any conflicts were resolved by independent experts (S.R.M. and H.M.-R.).

### 2.5. Data Items

The following data were extracted from the included articles: bibliographic details of articles (name of authors and publication year), aim of the study, data source, data structure and size (train/valid/test), data preprocessing, applied models, applied deep learning model, and reported results.

### 2.6. Study Risk of Bias (ROB) Assessment

The modified QUADAS-2 tool was used for critical appraisal based on the protocol mentioned in our previous studies [6, 29]. Two investigators (M.Y. and M.N.) performed the analyses, and any disagreements were solved by independent experts (S.R.M. and H.M.-R.). Studies in which the function of the AI models was compared to a defined reference were considered for the ROB assessment. Concerning the reference standard domain of the ROB assessment, the methodology of each article was individually assessed to define a reference standard based on validity and reliability, in addition to feasibility and accessibility of that reference. In this process, alternative references were considered, and the best one was introduced as the reference standard. It is noteworthy that this process was done by several authors, including one orthodontist specialist.

### 2.7. Synthesis of Results

Considering the heterogenicity of the studies, meta-analyses were not performed. Meanwhile, the distribution of the studies based on their aim, publication year, and applied AI models was reported qualitatively.

## 3. Results

### 3.1. Study Selection

**Figure 8:**
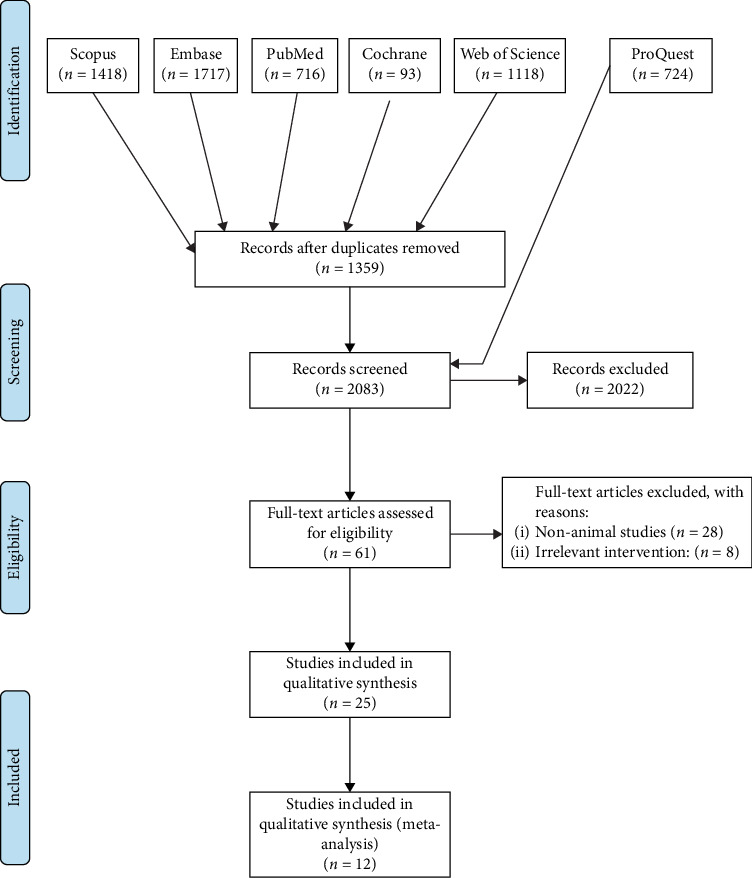
Flow diagram.

**Figure 9:**
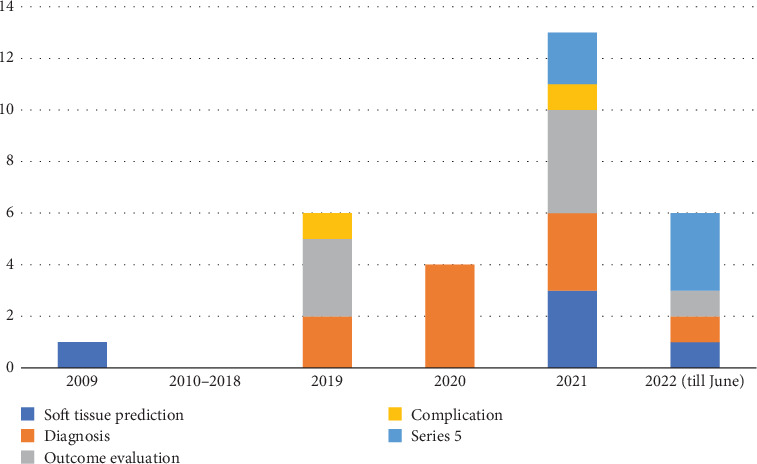
Distribution of the studies based on their publication year.

From 656 studies, a total of 29 articles met the inclusion criteria and were included in the study (Figure 8). Included studies have been published from 2009, with an 8-year gap between 2010 and 2018 in which no studies were published (Figure 9). Although the main focus of the studies was on the diagnosis procedure, an increasing trend in treatment planning studies can be seen.

### 3.2. Study Characteristics

A total of 10 studies fit into the diagnosis group, five into treatment planning, five into soft tissue prediction, seven into the outcome evaluation, and two into the complication assessment category (Tables 2, 3, 4, 5, and 6). Knoops et al. [21] and Khosravi-Kamrani et al. [17] were the only studies that used AI in both diagnosis procedures and analyzing the outcome of the surgery. Moreover, only Knoops et al. [21], Dot et al. [34], and Patcas et al. [47] used public datasets, while others used private datasets. The most common source of the dataset was from South Korea. In the diagnostic group, the aim of the included studies can be categorized as follows:

1. Defining the need for orthognathic surgery [11, 21, 26, 30–32]
2. Patient classification [17]
3. Predicting the need for future orthognathic surgery in cleft patients [18, 23]
4. Automatic registration of occlusal scans and computed tomography (CT) data [33]

In the treatment planning group, the aim of the included studies can be categorized as follows:

1. Osteotomy design [25]
2. Segmentation of surgical site in CT images [34]
3. Postoperative skeletal change prediction [35]
4. Bony reference definitions (44, 45)

For soft tissue prediction, the aim of the included studies can be categorized as follows:

1. Comparison of AI with finite element method (FEM) [3, 38]
2. AI with mass tensor model (MTM) [37]
3. Improving the function of the video imaging [40]
4. Developing an AI system for soft tissue prediction [39]

In the outcome evaluation group, the aim of the included studies can be categorized as follows:

1. Compare landmark alternations in two surgical approaches [41]
2. Compare pre- and postsurgical soft tissue [42]
3. Evaluate the alternations in facial asymmetry following surgery [43–45]
4. Analyze the impact of surgery on facial attractiveness [46, 47]

In the complication assessment group, the aim of the included studies can be categorized as follows:

1. Predict the blood loss [22]
2. Postsurgical infections [48]

**Figure 10:**
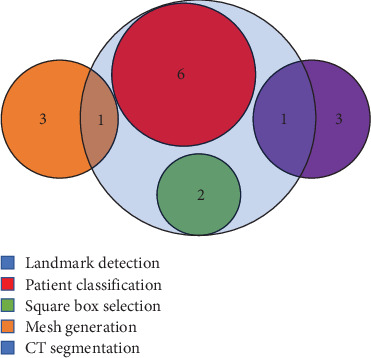
Venn diagram of the preprocessing procedure of the included studies based on the count of the study.

**Figure 11:**
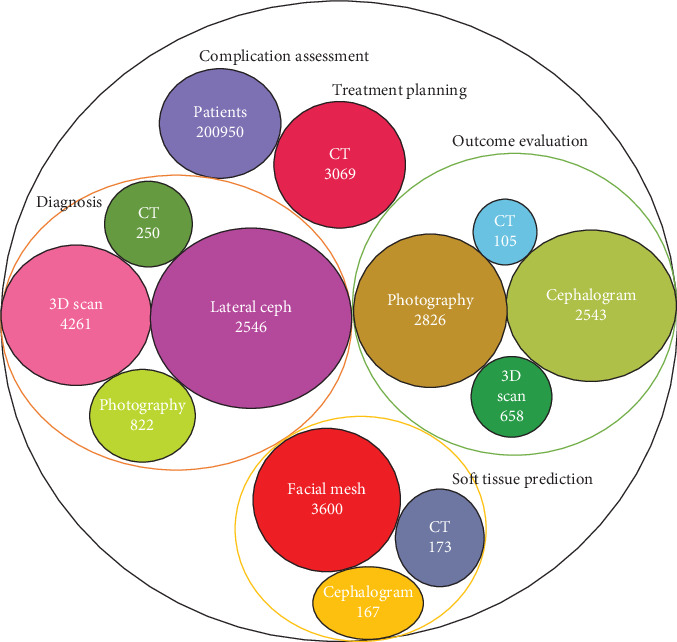
Data modality that is used in the included studies. Size of the circles is based on the count of the patients.

A total of five [17, 18, 21, 23, 42] of the included studies did not use deep learning models for their analyses. Among the 23 studies that used deep learning methods, 11 used CNN, three used ANN, one used AE, two used both ANN and AE, and six did not specify their deep learning method. Preprocessing methods and data modalities that were used in the included studies are shown in Figures 10 and 11.

### 3.3. ROB Within Studies

Table 7 contains detailed information on the ROB of the studies. Only six studies had a low ROB; among them, three were related to the diagnosis category (33.34%) [11, 30, 31], two to treatment planning (40%) [25, 36], and one to soft tissue prediction (25%) [39]. The reference standard was the most common high-risk factor in the included studies, while all of the studies were at low ROB for flow and timing.

### 3.4. Results of Individual Studies

#### 3.4.1. Diagnosis

Among the studies that analyzed the need for surgery, Knoops et al. [21] reported the highest sensitivity of 95.5% based on the 4261 3D surface scans imported into their designed 3D morphable model. Meanwhile, the highest specificity belonged to CNN models used in a study by Shin et al. [30], in which 607 cephalometry images were used for analyses. On the other side, the lowest specificity was 91.7%, related to a study by Jeong et al. [32] in which 822 facial photography images were used. Moreover, the lowest sensitivity (i.e., 75%) was related to the ResNet 50 model used in a study by Lee et al. [31], in which 333 lateral cephalometry images were used for analyses.

Khosravi-Kamrani et al. [17] used AI to classify the Class III patients and showed that orthognathic surgery is most commonly required in patients with mandibular prognathism and least in patients with combined maxillomandibular issues. Besides, Lin et al. [23] reported that AI models were able to use lateral cephalometry images at age 6 to predict the need for future orthognathic surgery with an accuracy of 87.4%. AI models also showed an accuracy of 33.09% in combining the occlusal scan and CT data in the diagnosis procedure [33].

#### 3.4.2. Treatment Planning

Qiu et al. [25] demonstrated that the safety, symmetry, and esthetic indices for presurgical planning of mandibular angle osteotomy were better recognized using CNN. Besides, CNNs were able to accurately detect the landmarks on the CT images and predict their postoperative situation based on the imported osteotomy designs with an average error of 5 mm [35]. Moreover, in the asymmetry cases, deep learning has also been used to design the osteotomies in the intended site based on the data acquired from the opposite facial area. It has been shown that SDNet and DefNet were the most accurate DL models to define the bony reference points in the treatment planning phase [12, 36].

#### 3.4.3. Soft Tissue Prediction

Among the studies of this category, two aimed to compare the deep learning model with FEM using facial mesh [3] or CT images [38] and showed that AI has comparable accuracy with FEM for soft tissue prediction while the procedure performed faster using AI. The rest of the studies analyzed the function of their proposed models in predicting soft tissue following mandibular advancement and bimaxillary orthognathic surgeries. Tanikawa and Yamashiro [39] reported a 100% success rate for their DL model based on 137 lateral cephalometric and 3D facial images, while the least reported success rate was for Ter Horst et al. [37] (i.e., 92.9%), who used 133 CBCT scans and 3D facial images to analyze the function of their ANN and AE models.

Besides, Ter Horst et al. [37] reported that their models had higher levels of accuracy in predicting the lower lip region compared to the chin in the mandibular advancement surgery. However, Lu et al. [40] mentioned that the lower lip was the least predictable component in the bimaxillary surgery. The former study was based on 133 CBCT images and 3D photographs, while the latter was based on 30 lateral cephalograms and profile photographs. Additionally, Lu et al. [40] reported that soft tissue prediction accuracy was higher in the nose tip and upper lip compared to the other areas of the maxilla.

#### 3.4.4. Outcome Evaluation

ANN and AE models were used to compare the pre- and postsurgical differences in the skeletal landmarks between the surgery first and traditional approaches using 2843 cephalometry images and showed acceptable results for the former in treating patients with facial asymmetry [41]. Seo et al. [42] used AI to evaluate the soft tissue changes in cleft patients who underwent bimaxillary surgery. Besides, Lin et al. [43, 44] and Lo et al. [45] used AI models to evaluate the facial asymmetry quantitatively to analyze the efficacy of the intervention and showed improved results following orthognathic surgery. Facial attractiveness was the other factor that was quantitatively analyzed by AI and improved significantly following orthognathic surgery [46, 47].

#### 3.4.5. Complication Assessment

Allareddy et al. [48] declared that the ANN model predicts systemic infections following orthognathic surgeries better than statistical methods. The accuracy of 98.7% and the AUC of 0.87 for the AI model are reported in this study based on the data obtained from 200 patients. Additionally, Stehrer et al. [22] used the random forest model to predict postoperative blood loss and found a strong link between actual and predicted blood loss. Data from 900 patients who underwent orthognathic surgery was used in the mentioned study.

## 4. Discussion

This study systematically reviewed articles in which AI was used in orthognathic surgery patients. Although AI does not decrease the costs significantly, it has fastened the procedure to some extent (i.e., it requires several thousands of milliseconds) while having a proper accuracy [49]. Results of the included studies showed that AI has a proper function in different fields of orthognathic surgery.

ML in surgeries has evolved from retrospective risk prediction and diagnostics to real-time intraoperative decision-making and AI-assisted robotics. Recent advancements, such as transformer-based architectures and multimodal AI, enhance precision and clinical integration, shaping the future of AI-driven surgeries [50].

Studies showed that AI was able to define the need for surgery with high levels of accuracy. Meanwhile, it must be considered that the data modality on which the model was trained can impact the accuracy of the diagnosis procedure. Since both esthetic and nonesthetic issues can be the reason for surgery, the imported data must be from soft tissue and hard tissue scans. For instance, importing only facial photographs leads to focusing only on the esthetic aspect and therefore missing 12.5% of the patients who have to undergo orthognathic surgery [21]. To increase the accuracy, multiple types of data can be imported. For instance, Shin et al. [30] imported both posterior–anterior and lateral cephalometry data to increase the accuracy of the machine as well as make the model useful in treatment planning for patients with facial asymmetry.

One interesting reported application of AI was predicting the need for further orthognathic surgery in patients with cleft lip and palate. This incident is based on several factors, including cleft type, the severity of lip separation at birth, the number of missing teeth, sex, surgery methods of palatal closure, duration of orthodontic treatment, and data acquired from lateral cephalometry. Analyzing all of these data together can be accurately and quickly performed by computer-based models such as those used in AI. Lim et al. [18] performed the analyses based on the evaluations performed when patients were 7 years old and showed a sensitivity of 78%. Considering that orthodontic treatments usually begin at 5–7 years old, these data can be helpful in treatment planning and reducing deterioration.

Considering the anatomical structures of the maxillofacial region, nerve/vessel damage may occur during osteotomizing maxillofacial bones. Computer-aided methods such as AI can help to overcome this issue. Qiu et al. [25] showed that their AI models created precise osteotomies that were safe and provided the optimal symmetry in the esthetic regions. However, their results were limited to mandibular osteotomy design. On the other side, Xiao et al. [12] proposed a deep learning model with proper accuracy in which a bony reference is defined automatically, and the required bony movement is demonstrated to reach the reference bony shape.

Virtual surgical planning systems are commonly used in the treatment planning phase of orthognathic surgeries. However, complexity, contradicting accuracy, and time-consuming procedures (i.e., about 25 min) make researches to find methods to improve these systems [12, 25]. Additionally, using VSP models, surgeons must analyze different treatment approaches and osteotomies and choose the optimal one. However, AI can make treatment planning a single-step fully automated process in which the optimal osteotomy is recommended, and the related soft tissue is presented simultaneously [21]. Yet, it is still the responsibility of the practitioner to decide whether the final soft tissue caused by the osteotomy is appropriate or not. Included studies showed accurate osteotomy design with proper safety for the AI models [21, 25].

AI models are not able to recommend the optimal osteotomy to reach the required soft tissue. Indeed, a mathematical method is required to inversely define the position of the maxillomandibular complex from the skull based on the defined soft tissue [51]. A model that can perform combined soft and hard tissue analyses may help overcome the issue [52]. However, mentioned solutions require large CT and MRI datasets that are not currently available. None of the included studies has proposed such a model.

Predicting the postsurgical soft tissue using the commonly available software is time-consuming and requires varied data to be imported into the system [3, 53]. However, the included studies showed that AI was able to perform this task in a shorter time with comparable accuracy [3, 37]. However, the nonlinear relationship between the soft tissue and bony movements complicates the soft tissue prediction following orthognathic surgery, even for AI-based models [54]. For instance, Ter Horst et al. [37] mentioned that in the chin region, the accuracy of the baseline images might be impaired and impact the final prediction since the complete relaxation of the mentalis muscle may not be feasible during 3D imaging. Moreover, the mentioned study [37] reported that in the lower lip, a commonly used algorithm is considered a behavior for the lip based on its attachment to the hard tissue while it has a sliding function rather than strict attachment [55]. Tanikawa and Yamashiro [39] reported that the upper lip, nasal base, and angle of the mouth are the maxillary structures that can complicate the prediction procedure. In addition to the mentioned point, the accuracy of the models decreased when considering 1 mm errors as unsuccessful outcomes. Therefore, more accurate models are still required for soft tissue prediction.

AI has been widely used to evaluate the impact of orthognathic surgery on facial appearance. Indeed, quantitative facial asymmetry and attractiveness analyses are pragmatic with AI models [56]. These results can be used in research to evaluate the efficacy of surgical procedures and to increase the patient's information about the necessity of the surgery. For instance, Patcas et al. [47] reported a more significant impact for mandibular surgeries on facial appearance than maxillary displacements and a 64% improvement in facial appearance after the intervention.

Predicting surgical complications, including blood loss and severe infection, is a beneficial task that AI models can perform. The provided data can be helpful in patient selection and evaluating the risk of the surgery. Besides, the data regarding the evaluated risk can be provided to insurance companies to update their policies based on the cost-effectiveness of each surgical intervention. It has to be considered that the data-gathering phase to treat the machine for this purpose is less complicated than those procedures related to diagnosis and treatment planning. Therefore, studies with larger sample sizes can be performed, which may lead to creating models with higher levels of reliability.

Methods using AI have been shown to improve accuracy by analyzing huge amount of data and more precise measurements compared to conventional methods. In addition, AI improves the efficacy of treatments and clinical outcomes by planning surgeries, predicting soft tissue changes, and forecasting complications. Larger datasets lead to better accuracy, and an insufficient quantity of datasets leads to overfitting, which happens when the model performs well on training datasets but poorly on new and unseen ones. However, after reaching a certain point on quantity, adding more datasets leads to minimal accuracy gains, especially when datasets are similar to the previous ones. Moreover, relevant, high-quality, balanced, and diverse datasets help the model learn meaningful patterns. On the other hand, poor-quality datasets with irrelevant information mislead the model.

AI can be integrated into daily clinical practice by being used in preoperative assessment, treatment planning, intraoperative guidance, and postoperative monitoring. AI can further be used to improve orthognathic surgeries in following ways: enhance the accuracy of interpreting diagnostic images, improve the precision of surgical procedures by generating 3D models based on patients' anatomies and design the best and less traumatic movements, analyze data gathered from patients to predict recovery time, assess probable complications, and create personalized treatment plans.

### 4.1. Limitations and Recommendations

Including samples had relatively restricted inclusion criteria and trained the models based on their limited included patients. Therefore, the models may not be comprehensive enough, and their accuracy may be decreased when importing patients with features other than those used to train the model. Additionally, the imaging setup, data gathering, and labeling are factors that significantly impact the models' function. Moreover, the soft tissue response to the hard tissue movement can be affected by age, ethnicity, tissue texture, gender, and the presence of orthodontic appliances. Therefore, it is essential to perform more multicenter studies with high counts of variable samples to analyze the model's generalization. Consequently, considering the significant positive impact of using large datasets in training the algorithm, it is recommended to establish an online database in which the preoperative data and the results are uploaded. Another significant challenge is the clinical integration of ML models, as their adoption is often hindered by workflow incompatibility, physician skepticism, and the need for regulatory approvals. Additionally, the lack of standardized validation protocols across different surgical settings further complicates real-world implementation. The reliability of the outcome generated from the machine is significantly related to the method used to train the model. Indeed, the machine may perform accurately based on the defined ground truth. However, the ground truth itself may be inaccurate. In issues such as soft tissue prediction, importing the pre- and postsurgical data can provide accurate ground truth. On the contrary, to define the need for surgery and treatment planning, a group of experts is required to perform accurate analyses and import the correct data to the system based on the case analyses, radiographic images, and clinical evaluations.

Two of the categories (outcome evaluation and complication assessment) contained no study with low ROB. This result indicates the need for improvements in further studies, especially in these categories. In addition, different methodologies of studies need different ways of assessing ROB, by which we could not make a comprehensive comparison. Thus, one kind of ROB assessment was used in our study.

Regarding patient selection domain, soft tissue prediction category is more prone to high ROB than other categories (75%). To teach the ML model how to predict the result of soft tissue in patients undergoing orthognathic surgeries, before and after images of training datasets are given to the model. Consequently, high ROB in patient selection might be because researchers tend to include patients with more typical and acceptable results in their studies. While it is more plausible to include all patients randomly to let the ML model learn and foresee all kinds of soft tissue results. Regarding the index test domain, treatment planning, outcome evaluation, and soft tissue predictions had higher ROB than other categories (100%), which could be avoided by fully reporting the ML model description and accuracy, specificity, and sensitivity of the models.

Reference standard was the most common high-risk factor in included studies. Regarding different categories, the diagnosis category is more prone to high ROB in reference standard (88.89%). This could be because in the diagnosis stage, less objective and reliable information is available, and diagnosis is based on the subjective view of the clinician more than other stages. But to include datasets in the studies, patient information should be more defined and objective.

Flow and timing domain indicates how time affects ML model assessment, which is not reliable in our study. High ROB increases model inaccuracies and leads to over- or underestimated results. Indeed, not only does high ROB affect original studies but it also affects our review. In our review, uncertainty and inconsistency of results happen when original articles lack certainty due to high ROB. Our review suggests that future studies should pay attention to minimizing the ROB by using plausible inclusion and exclusion criteria; defining test, train, and valid datasets if the study is not cross-validation; using randomized dataset selection for patient selection; indicating model description and accuracy, sensitivity, and specificity for the index test; and an objective reference standard for the reference standard.

## 5. Conclusion

AI models have been successfully used in the diagnosis, treatment planning, soft tissue prediction, outcome evaluation, and complication assessment phases of orthognathic surgery. However, more multicenter studies with higher sample sizes are required to confirm the generalization of the results. Additionally, more accurate methods must be created to define the ground truth of the models to decrease the concerns regarding the accuracy of the procedures. Public databases must be created to increase the data availability and help reach the mentioned goals.

## Figures and Tables

**Table 1:** Search queries used in the mentioned databases.

**Database**	**Search query**	**Result**
PubMed	(“artificial intelligence”[MeSH Terms] OR “deep learning”[MeSH Terms] OR “machine learning”[MeSH Terms] OR “artificial intelligence” OR “deep learning” OR “machine learning” OR “neural network” OR “diagnosis, computer assisted”[MeSH Terms]) AND (lefort osteotomy[MeSH Terms] OR orthognathic surgery[MeSH Terms] OR orthognathic surgical procedure[MeSH Terms] OR “maxillary advancement” OR BSSO OR IVRO OR SSRO OR IVSRO OR ((maxilla OR mandibl⁣^∗^ OR mandibular) AND (surg⁣^∗^ OR osteotom⁣^∗^ OR reposition⁣^∗^ OR re-position OR section OR advance⁣^∗^ OR setback OR set-back)))	534
Google Scholar	Allintitle:(“artificial intelligence” OR “deep learning” OR “machine learning” OR “neural network”) AND (“lefort osteotomy” OR “orthognathic surgery” OR “maxillary advancement” OR “mandibular advancement” OR “maxillary setback” OR “mandibular setback”)	8
Scopus	TITLE-ABS-KEY ((“artificial intelligence” OR “deep learning” OR “machine learning” OR “neural network”) AND (lefort OR “orthognathic surgery” OR “orthognathic surgical procedure” OR “maxillary advancement” OR bsso OR ivro OR ssro OR ivsro OR ((maxilla OR mandible OR mandibular) AND (surg⁣^∗^ OR osteotom⁣^∗^ OR reposition⁣^∗^ OR re-position⁣^∗^ OR section OR advance⁣^∗^ OR setback OR set-back))))	138
Cochrane Library	(“artificial intelligence” OR “deep learning” OR “machine learning” OR “neural network”) AND ((“maxillary advancement” OR (maxilla OR mandibl⁣^∗^ OR mandibular) AND (surg⁣^∗^ OR osteotom⁣^∗^ OR reposition⁣^∗^ OR re-position OR section OR advance⁣^∗^ OR setback OR set-back)))	17
Web of Science	((((TS = (artificial intelligence)) OR TS = (deep learning)) OR TS = (neural network)) OR TS = (machine learning)) AND (((((((((TS = (orthognathic surgery)) OR TS = (maxillary advancement)) OR TS = (lefort osteotomy)) OR TS = (BSSO)) OR TS = (mandibular reposition)) OR TS = (maxillary reposition)) OR TS = (mandibular advancement)) OR TS = (maxillary setback)) OR TS = (mandibular setback))	55
Embase	(“artificial intelligence”/exp OR “artificial intelligence” OR “deep learning”/exp OR “deep learning” OR “machine learning”/exp OR “machine learning” OR “neural network”/exp OR “neural network”) AND (“lefort osteotomy”/exp OR “lefort osteotomy” OR “orthognathic surgery”/exp OR “orthognathic surgery” OR “maxillary advancement”/exp OR “maxillary advancement” OR “mandibular advancement”/exp OR “mandibular advancement” OR “maxillary setback” OR “mandibular setback”/exp OR “mandibular setback”)	65

**Table 2:** Result of the studies analyzing the application of AI in the diagnosis phase.

**Author(s) (year)**	**Aim**	**Dataset source**	**Dataset size and structure (train/valid/test)**	**Data preprocessing**	**- Applied method category** **- Model name**	**DL**	**Outcomes**
Mohammad-Rahimi et al. (2021) and Shin et al. (2021) [6, 30]	- Need for orth surgery in Classes 2 and3 and asymmetry	Private dataset	607 cephalograms (273/30/304)	- Landmark detection - Patient classification	ResNet: - ResNet 34	+ CNN	Accuracy: 95.4% Sensitivity: 0.884 Specificity: 0.993

F. Jiang et al. (2017) and Kim et al. (2021) [7, 11]	- Need for orth surgery comparing different AI models	Private dataset	960 cephalograms (NM/810/150) (640 nonsurgical and 320 surgical) Five-fold cross-validation was performed	- Landmark detection - Minimum and square box selection	ResNet: - ResNet 18 - ResNet 34 - ResNet 50 - ResNet 101	+ CNN	AUC: - ResNet 18: 0.97 - ResNet 34: 0.97 - ResNet 50: 0.945 - ResNet 101: 0.944 Accuracy: - ResNet 18: 93.80% - ResNet 34: 93.60% - ResNet 50: 91.13% - ResNet 101: 91.33% Sensitivity: - ResNet 18: 88.2% - ResNet 34: 87.6% - ResNet 50: 80.6% - ResNet 101: 82.4% Specificity - ResNet 18: 96.6% - ResNet 34: 96.6% - ResNet 50: 96.4% - ResNet 101: 95.8%

Kharnagar et al. (2021) and Lee et al. (2020) [9, 31]	- Need for orth surgery using lateral cephalometry	Private dataset	333 lateral cephalograms (220/73/40) Four-fold cross-validation was performed	- Landmark detection - Minimum and square box selection	AlexNet: - Modified AlexNet - MobileNet ResNet: - ResNet 50	+ CNN	AUC: - Modified AlexNet: 0.96 - MobileNet: 0.90 - ResNet 50: 0.92 Accuracy: - Modified AlexNet: 96.4% - MobileNet: 95.4% - ResNet 50: 95.6% Sensitivity: - Modified AlexNet: 85.2% - MobileNet: 76.1% - ResNet 50: 75% Specificity: - Modified AlexNet: 97.3% - MobileNet: 93.1% - ResNet 50: 94.4%

Kim et al. (2021) and Choi et al. (2019) [11, 26]	- Need for orth surgery or dental extraction	Private dataset	316 cephalograms (136/68/112)	- Landmark detection - Patient classification	NM	+ ANN	- Success rate of surgery/nonsurgery diagnosis for total: 96% - Success rate of Classes II and III surgical type classification: 100% - The success rate of extraction/nonextraction diagnosis for Classes II and III: 97% and 88%, respectively - The final diagnosis success rate: 91%

Cortes and Vapnik (1995) and Jeong et al. (2020) [20, 32]	- Need for orth surgery based on facial photography	Private dataset	822 facial photographs of front and right Side (411/NM/411) (411 nonsurgical and 411 surgical)	- Landmark detection - Patient classification	VGGNet: - VGG19	+ CNN	Accuracy: 89.3% Precision: 91.2% F1 Score: 88.9% Sensitivity: 86.7% Specificity: 91.7%

Xiao et al. (2021) and Knoops et al. (2019) [12, 21]	- Diagnosis and outcome - Need for orth surgery based on 3D face scans	Public/private dataset	4261 3D surface scan Monte Carlo cross-validation Scheme was performed	- Mesh generation	- 3D morphable models: For classification: - SVM For regression: - Linear regression - Ridge regression - Least-angle regression - Least absolute shrinkage - Selection operator regression	—	Sensitivity: 95.5% Specificity: 95.2% Positive predictive value: 87.5% Negative predictive value: 98.3%

Lampen et al. (2022) and Khosravi-Kamrani et al. (2022) [3, 17]	- Diagnosis and outcome - Define the Class 3 subtype and evaluate the prevalence of orth surgery using AI	Private dataset	148 lateral cephalograms	- Patient classification - Landmark detection	SPM: - SPM3 Distance weighted discrimination (DWD)	—	NM
							- Likelihood of surgery and treatment failure: Mandibular prognathism > maxillary deficiency > combination - Treatment failure: Nonsurgical > surgical

Thrall et al. (2018) and Lin et al. (2020) [8, 23]	- Predicting the need for future orth surgery in cleft patients	Private dataset	56 lateral cephalograms Ten-fold cross-validation was performed	- Patient classification - Landmark detection	Decision trees - XGBoost (to determine the need for orth surgery) - Random forest - Boruta (for cephalometric predictions at T0)	—	Accuracy: 87.4% Sensitivity: 97.83% Specificity: 90.00% *F*1-score: 0.714

LeCun et al. (2015) and Lim et al. (2021) [18, 24]	- Define the prognostic factors of orth surgery in cleft patients - Predict the future need for orth surgery, skeletal discrepancy, and revere overjet	Private dataset	Lateral cephalograms of 126 patients with CL/P	- Landmark detection - Patient classification	Logistic regression	—	- Need for orth surgery: AUC: 0.9–0.91 Sensitivity: 0.78 Specificity: 0.87 - Skeletal discrepancy: AUC: 0.9 Sensitivity: 0.96 Specificity: 0.88–0.92 - Overjet AUC: 0.9 Sensitivity: 0.78–0.89 Specificity: 0.87–0.90
						—	Palatal closure: Push back method > Furlow's method

Choi et al. (2019) and Chung et al. (2020) [26, 33]	- Combining the CT data and occlusal scan for orth surgery	Private dataset	250 patients, 250 CTs, and 500 scanned models (100 CTs and 200 scanned models, 150 CTs and 300 scanned models, NM) Three-fold cross-validation was performed	- Landmark detection	A combination of deep pose regression and optimal cluster-based matching	+ CNN	Registration accuracy: 33.09%

**Table 3:** Result of the studies analyzing the application of AI in the treatment planning phase.

**Author(s) (year)**	**Aim**	**Dataset source**	**Dataset size and structure (train/valid/test)**	**Data preprocessing**	**- Applied method category** **- Model name**	**DL**	**Outcomes**
Kaul et al. (2020) and Qiu et al. (2022) [1, 25]	- Osteotomy design in mandibular angle orth surgery	Private dataset	2296 normal 3D CTs (1900/396/NM-)	- Feature extraction - Segmentation	Pointcloud: - 3D point cloud model - Nonequivalent point network (NEPN)	+ CNN	- Safety: AI > manual - Asymmetry: Mandibular angle: AI > manual Mandibular ramus height: AI < manual - Esthetic: Width ratio between middle and lower face: AI < manual Mandibular angle: AI > manual Valgus angle: AI > manual

Chowdhury and Sadek (2012) and Dot et al. (2022) [4, 34]	- CT segmentation in orth surgery	Public/private dataset	453 CT images (300/10/153) Five-fold cross-validation was performed	- CT segmentation	- U-Net: - nnU-Net	+ AE	- Industry expert segmentation approval rates: 42.2% in total 75.6% in total without teeth mask - Surface dice similarity coefficient at 1 mm: 98.03% ± 2.48% - Volumetric dice similarity coefficient: 92.24 ± 6.19

Holzinger et al. (2019) and Ma et al. (2022) [10, 35]	- Predicting the postoperative skeletal changes	Private dataset	56 CT images (50/NM/6)	- Landmark detection		+ CNN	Accuracy: Landmark level: 5.4 ± 0.6 mm Volume level: 74.4%

Peters et al. (2015) and Xiao et al. (2021) [28, 36]	- Bony reference definition	Private dataset	132 CTs (108/NM/24)	- CT segmentation - Mesh generation - Landmark detection	- Sparse representation (SR) - Landmark-based sparse representation (LSR) - Surface deformation network (SDNet) - Pointcloud - Surface deformation network (SDNet) - Point-Net-Reg - CPD-Net	+	- Accuracy Jaw: SDNet > LSR SDNet > CPD-Net VD, SC, and LD: SDNet = Point-Net-Reg ED: SDNet > Point-Net-Reg Midface: Comparable Ical acceptance: SDNet > LSR
Mohammad-Rahimi et al. (2021) and Xiao et al. (2021) [6, 12]	- Bony reference definition	Private dataset	132 CTs (108/NM/24) Nine-fold cross-validation was performed	- CT segmentation - Landmark detection	- Sparse representation (SR) - Pointcloud - Point-Net-Reg - PointNet++ - Deformation network (DefNet) - RSCNN - KPConv - RandLA-Net	+	- Accuracy: Based on synthetic patients: Comparable Based on real patients: DefNet > SR Midface and jaw region for both models: Comparable - Accuracy: Jaw: DefNet > alternative networks Midface: DefNet, PointNet++, RSCNN equal > KPConv, and RandLA-Net

**Table 4:** Results of the studies analyzing the application of AI in soft tissue prediction phase.

**Author(s) (year)**	**Aim**	**Dataset source**	**Dataset size and structure (train/valid/test)**	**Data preprocessing**	**- Applied method category** **- Model name**	**DL**	**Outcomes**
Shan et al. (2021) and Ter Horst et al. (2021) [13, 37]	- Compare AI with MTM BSSO	Private dataset	133 CBCT scans and 3D facial images (119/NM/14)	- Landmark detection - Mesh generation - Facial mesh matching from T1 to T2	- DL-based - Keras - Mass tensor–based model - IPS CaseDesigner	+ ANN AE	Success rate: 64.3% (high degree) 92.9% (medium degree) AI > MTM Error: lower lip < chin region

Xu et al. (1999) and Lampen et al. (2022) [2, 3]	- Comparing FEM with DL	NM	Facial mesh, input displacement, and explicit boundary type information (70%, 10%, and 20%) (total NM)	- Facial mesh generation	- Pointcloud: - PointNet++	+	- Mean error: 0.159–0.642 mm - Including explicit boundary had contradicting results

Khosravi-Kamrani et al. (2022) and Ma et al. (2021) [17, 38]	- Comparing FEM with DL	NM	40 paired head CT scans (pre- and post-op) (32/NM/8) Five-fold cross-validation was performed	- CT segmentation - Landmark detection - Region of interest extraction	- Pointcloud - FC-Net	+	- Average landmark error: with (3.15 mm) and without self-attention module training (3.26 mm) - AI faster than FEM-RLSE

Choi et al. (2020) and Tanikawa and Yamashiro (2021) [14, 39]	- Prediction in bimaxillary orth surgery GMM and DL-incorporated system expansion for 3D facial morphology anticipation after orth surgery and fixed orth tx	Private dataset	137 lateral cephalograms and 3D facial images Eleven-fold cross-validation was performed	- Landmark detection	- System S	+	- System error: 0.94 ± 0.43 - Success rate 74% (high degree) 100% (medium degree) - Complexity: System S > commercial - Software program error: System S < commercial - Software program error: Lower lip < nasal alar, chin, and corner of the mouth

Lin et al. (2021) and Lu et al. (2009) [23, 40]	- Improving video imaging in bimaxillary protrusion	Private dataset	30 lateral cephalograms and profile photographs (60/NM/NM)	- Landmark detection	NM	+ ANN	- Accuracy: Tip of the nose and upper lip > other areas > lower lip - Prediction error improvement rates: Sagittal > vertical

**Table 5:** Results of the studies applying AI in outcome evaluation phase.

**Author(s) (year)**	**Aim**	**Dataset source**	**Dataset size and structure (train/valid/test)**	**Data preprocessing**	**- Applied method category** **- Model name**	**DL**	**AI-related outcomes**	**Patient-related outcomes**
Nichols et al. (2019) and Choi et al. (2022) [5, 41]	- Comparing the changes on the landmarks between two approaches	NM	2843 cephalograms (2275/284/284)	NM	Region of interest detection: - RetinaNet Landmark detection: - U-Net	+ ANN AE	- Interrater reliability: 0.90	- Surgery first had no ss diff with traditional approach

Schwendicke et al. (2020) and Seo et al. (2021) [15, 42]	- Pre–post soft tissue comparison after bimaxillary surgery in cleft	Private dataset	34 3D CTs (16 control and 18 experimental)	NM	- ON3D	—	NM	CLP patients: More posterior tissue landmarks, a larger alar and philtrum width, and a less sharper nasal tip angle

Nasteski (2017) and Lin et al. (2021) [16, 43]	- Evaluating the facial asymmetry	Private dataset	71 CBCT images (NM)	- Contour map extraction - Feature enhancement - Data amplification	- VGGNet - VGG16 - VGG19 - ResNet - ResNet 50 - Inception - Xception	+ CNN	Accuracy: - VGG16: 80% - VGG19: 86% - ResNet 50: 83% - Xception: 90%	NM

Stehrer et al. (2019) and Lin et al. (2019) [22, 44]	- Evaluating the pre–post facial asymmetry and specify the impact of orth surgery	Private dataset	500 3D facial images (350/75/75) (70%/15%/15%)	- Facial symmetry rating - Region of interest extraction - Contour map extraction	NM	+ CNN	Accuracy: 2D: 65% 3D: 78.85% DLS > conventional 2D approaches	Degree of facial symmetry (mean): - Pre-op: 0.92 ± 0.17 - Post-op: 0.65 ± 0.13

Lim et al. (2021) and Lo et al. (2021) [18, 45]	- Comparing pre–post facial soft tissue symmetry	Private dataset	158 three-dimensional facial photographs (NM)	- Region of interest extraction - Contour map extraction - Feature enhancement - Data amplification	- VGGNet - VGG16 - VGG19 - ResNet - ResNet 50 - Inception - Xception	+ CNN	NM	Asymmetry improvement: 21% ± 7%
Peng et al. (2002) and Peck et al. (2021) [19, 46]	- Compare pre–post facial attractiveness	Private dataset	65 patients (NM)	NM	- Haystack AI software	+	No ss diff between AI and human in evaluation	Attractiveness: Significantly improved

Knoops et al. (2019) and Patcas et al. (2019) [21, 47]	- Impact of orth surgery on facial attractiveness and age appearance	Public/private dataset	2761 photographs (469/128/2164)	- Patient classification	VGGNet: - VGG16	+ CNN	NM	- Age appearance: 66.4% improved ~1 year younger - Attractiveness: 74.7%

**Table 6:** Result of the studies analyzing the blood loss and systemic infection.

**Author(s) (year)**	**Aim**	**Dataset source**	**Dataset size and structure (train/valid/test)**	**Data preprocessing**	**- Applied method category** **- Model name**	**DL**	**AI-related outcomes**	**Patient-related outcomes**
Qiu et al. (2022) and Allareddy et al. (2021) [25, 48]	- Infection prediction	The nationwide inpatient sample	200 patient and hospital related variables (140/NM/60)	NM	Neural network compared with traditional multivariate logistic regression	+ ANN	Accuracy: 98.7% AUC: 0.87	Systemic infection rate: 1.1%

Moher et al. (2015) and Stehrer et al. (2019) [22, 27]	- Blood loss prediction	Private dataset	950 surgeries (760/NM/190)	NM	To predict blood loss: Random forest To compare predicted and actual values: Linear regression	—	Significant correlation between actual and predicted blood loss	NM

**Table 7:** Risk of bias of the included studies.

**Group**	**Study**	**Patient selection**	**Index test**	**Reference standard**	**Flow and timing**	**Overall**
Diagnosis	Shin et al. [30]	Low	Low	High	Low	Low
	Kim et al. [11]	Low	Low	High	Low	Low
	Lee et al. [31]	Low	Low	High	Low	Low
	Choi et al. [26]	Low	High	High	Low	High
	Jeong et al. [32]	High	Low	High	Low	High
	Knoops et al. [21]	High	Low	High	Low	High
	Lin et al. [23]	High	Low	High	Low	High
	Lim et al. [18]	High	High	High	Low	High
	Chung et al. [33]	Low	High	Low	Low	High

Treatment planning	Qiu et al. [25]	Low	High	Low	Low	Low
	Dot et al. [34]	High	High	Low	Low	High
	Ma et al. [35]	High	High	Low	Low	High
	Xiao et al. [36]	Low	High	Low	Low	Low
	Xiao et al. [12]	High	High	Low	Low	High

Soft tissue prediction	Ter Horst et al. [37]	High	High	Low	Low	High
	Lampen et al. [3]	High	High	Low	Low	High
	Ma et al. [38]	High	High	Low	Low	High
	Tanikawa and Yamashiro [39]	Low	High	Low	Low	Low

Outcome evaluation	Choi et al. [41]	Low	High	High	Low	High
	Seo et al. [42]	High	High	Low	Low	High
	Lin et al. [43]	Low	High	High	Low	High
	Lin et al. [44]	High	High	Low	Low	High
	Patcas et al. [47]	High	High	High	Low	High

Complication assessment	Allareddy et al. [48]	High	Low	High	Low	High

## Data Availability

No new data were generated or analyzed in this study. Data sharing is not applicable to this study.
